# GAHP: An integrated software package on genetic analysis with bi-parental immortalized heterozygous populations

**DOI:** 10.3389/fgene.2022.1021178

**Published:** 2022-10-05

**Authors:** Luyan Zhang, Xinhui Wang, Kaiyi Wang, Jiankang Wang

**Affiliations:** ^1^ National Key Facility for Crop Gene Resources and Genetic Improvement, and Institute of Crop Sciences, Chinese Academy of Agricultural Sciences (CAAS), Beijing, China; ^2^ Information Technology Research Center, Beijing Academy of Agriculture and Forestry Sciences, Beijing, China; ^3^ National Nanfan Research Institute (Sanya), Chinese Academy of Agricultural Sciences (CAAS), Hainan, China

**Keywords:** bi-parental population, immortalized heterozygous population, analysis of variance, QTL mapping, genetic simulation

## Abstract

GAHP is a freely available software package for genetic analysis with bi-parental immortalized heterozygous and pure-line populations. The package is project-based and integrated with multiple functions. All operations and running results are properly saved in a project, which can be recovered when the project is re-open by the package. Four functionalities have been implemented in the current version of GAHP, i.e., 1) MHP: visualization of genetic linkage maps; 2) VHP: analysis of variance (ANOVA) and estimation of heritability on phenotypic data; 3) QHP: quantitative trait locus (QTL) mapping on both genotypic and phenotypic data; 4) SHP: simulation of bi-parental immortalized heterozygous and pure-line populations, and power analysis of QTL mapping. VHP and QHP can be conducted in individual populations, as well as in multiple populations by the combined analysis. Input files are arranged either in the plain text format with an extension name same as the functionality or in the MS Excel formats. Output files have the same prefix name as the input file, but with different extensions to indicate their contents. Three characters before the extension names stand for the types of populations used in analysis. In the interface of the software package, input files are grouped by functionality, and output files are grouped by individual or combined mapping populations. In addition to the text-format outputs, the constructed linkage map can be visualized per chromosome or for a number of selected chromosomes; line plots and bi-plots can be drawn from QTL mapping results and phenotypic data. Functionalities and analysis methods available in GAHP help the investigation of genetic architectures of complex traits and the mechanism of heterosis in plants.

## 1 Introduction

In past decades, the methodology on quantitative trait locus (QTL) mapping has been extensively applied in genetic studies to dissect the individual genes of complex traits in both animals and plants. Bi-parental segregating populations, such as backcross (BC), doubled haploids (DH), recombinant inbred lines (RIL), and F_2_, are commonly developed and then used for QTL mapping studies in plants. A number of mapping methods have been proposed, such as interval mapping (IM; Lander and Botstein, 1989), composite interval mapping (CIM; Zeng, 1994), multiple interval mapping (MIM; Kao et al., 1999), inclusive composite interval mapping (ICIM; Li et al., 2007; Zhang et al., 2008), and multiple QTL model (MQM; van Ooijen, 2009). Some frequently used software packages for bi-parental populations are R/qtl (Broman et al., 2003), QTL Cartographer (Wang et al., 2007), QTLNetwork (Yang et al., 2008), MAPQTL (van Ooijen, 2009), and QTL IciMapping (Meng et al., 2015).

By comparison with the other mapping methods, ICIM is more efficient in background control *via* a two-step mapping strategy. In the first step of ICIM, stepwise regression is applied to identify the most-significant regression variables representing the marker genotypes. In the second step, interval mapping is performed on phenotypic values adjusted by marker variables identified in the first step (Li et al., 2007; Zhang et al., 2008; Meng et al., 2015). In recent years, the ICIM algorithm has been extended to epistatic mapping (Li et al., 2008a), QTL by environment interaction analysis (Li et al., 2015), hybrid F_1_ populations derived from two heterozygous parents, double cross F_1_ populations derived from four homozygous parents (Zhang et al., 2015a), and pure-line populations derived from four to eight homozygous parents (Zhang et al., 2017; Shi et al., 2019). The ICIM-based algorithms have been implemented in three integrated software packages, i.e. QTL IciMapping for bi-parental populations (Meng et al., 2015), GACD for hybrid F_1_ from two heterozygous parents and double cross F_1_ from four homozygous parents (Zhang et al., 2015b), and GAPL for multi-parental pure-line populations (Zhang et al., 2019).

Conventional heterozygous populations, such as BC, F_2_, and F_3_, may be used to estimate the dominance-related effects and investigate the genetic mechanism of heterosis. However, these populations cannot be phenotyped in multi-environmental trials, and thus the analysis for QTL stability and QTL by environment interaction cannot be conducted. To avoid these problems, the concept of immortalized F_2_ and BC has been proposed by using the bi-parental pure lines. For example, Hua et al. (2003) investigated the genetic basis of an elite rice hybrid using an immortalized F_2_ population by randomly permutated inter-mating of 240 bi-parental RILs. Liu et al. (2017) started from one RIL population of two maize inbred lines S-951 and Qi319, and developed one immortalized F_2_ population for QTL detection on leaf width. Yi et al. (2019) investigated the genetic bases of yield-related traits and heterosis in maize using immortalized F_2_ and RIL populations. Li et al. (2008b) reported two immortalized BC populations in rice and used them to identify the main-effect QTLs and digenic epistatic loci underlying the heterosis of agronomic and economic traits. Aakanksha et al. (2021) investigated the heterosis on yield in *Brassica juncea* by using a DH and two-directional immortalized BC populations. Li et al. (2018) developed two-directional immortalized BC populations and one immortalized F_2_ population, and used them to detect QTLs affecting fiber quality traits in upland cotton.

In studies mentioned above, immortalized heterozygous populations were treated as a kind of bi-parental populations in genetic analysis, and analyzed separately from their pure-line populations. The joint analysis of pure lines and their derived immortalized heterozygous populations provides more genetic information, and improves the mapping accuracy. In addition, no software package has been developed when heterozygous and pure-line populations are both available. In this study, we report an integrated software package which is called GAHP, i.e. genetic analysis with bi-parental immortalized heterozygous populations. By using this package, the phenotypic and genetic analysis can be performed in bi-parental immortalized populations and their pure lines either separately or jointly.

## 2 Materials and methods

### 2.1 Genetic mapping populations

**FIGURE 1:**
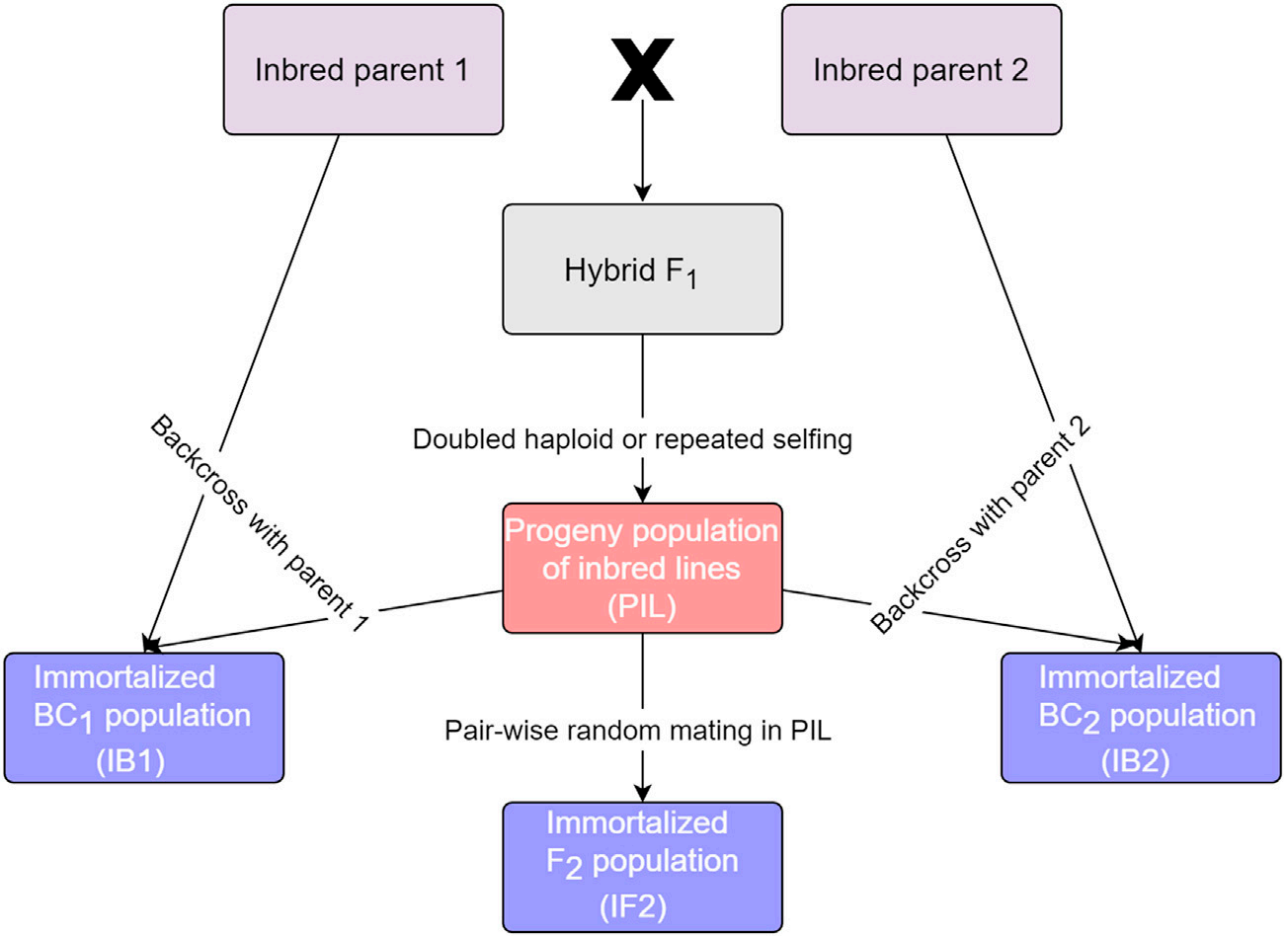
Relationship between populations that can be handled in GAHP.

Four kinds of populations, which are essentially derived from the same two homozygous parents, can be handled in GAHP for both phenotypic and genetic analysis. These populations are called by bi-parental pure-inbred lines (PIL), immortalized backcross population with the first parent (IB1), immortalized backcross population with the second parent (IB2), and immortalized F_2_ population (IF2). It should be noted that the pure-inbred lines (or pure lines in short) can be either DHs or RILs derived from two inbred homozygous parents. Relationship between the four populations is shown in Figure 1. Genotype of each line in population PIL can be maintained by selfing, which is the reason to be called ‘permanent’. IB1 is generated by the hybridization between the PIL population and the first inbred parent, similar to the backcrossing of F_1_ hybrid with the first inbred parent. IB2 is generated by the hybridization between the PIL population and the second inbred parent, similar to the backcrossing of F_1_ hybrid with the second inbred parent. IF2 is generated by the hybridization between two lines in the PIL population, similar to selfing of the F_1_ hybrid. As each line in population PIL can be maintained by selfing, IB1, IB2 and IF2 can be repeatedly produced like the typical F_1_ hybrids whenever needed, which is the reason to be called ‘immortalized’. Due to their repeatability, each of the four kinds of populations can be evaluated in multi-environmental trials with replications.

### 2.2 Coding criteria of marker types and phenotypic values

Both independent population and combined analysis can be conducted in GAHP. For genetic analysis, the genotypic data is only needed for population PIL. Genotypes of heterozygous lines in populations IB1, IB2, or IF2 can be deduced from the genotypes of homozygous lines in PIL. Assuming there are two homozygous parents P_1_ (or Parent A) and P_2_ (or Parent B), two bands can be observed in the two parents at one polymorphic marker locus. Markers having no polymorphism or heterozygous in either parent cannot be used. Assuming *AA* is the genotype of P_1_, *BB* is the genotype of P_2_, and *AB* is the genotype of their F_1_ hybrid. Marker types could be coded by numbers, letters, or the mixed numbers and letters. As individual lines in PIL are assumed to be homozygous, only homozygous genotypes in PIL are useful in genetic analysis. Heterozygous genotypes in PIL are treated as missing values. When numbers are used in coding, the two parental bands are coded as 2 and 0, respectively. When letters are used, Parent A is coded as A or AA; Parent B is coded as B or BB. Codes 1, H and AB are acceptable for heterozygotes, and missing values of marker types are coded as -1, X, XX, *, or **. Mixed coding with numbers and capital letters is allowed in the software, but it is not recommended. Missing phenotypic values are represented by “NA”, “na”, “*“, “.“, or “-100”, which will be replaced by population mean in QTL mapping.

### 2.3 Development of the GAHP software

In GAHP, core modules for phenotypic data analysis, QTL mapping, genetic population simulation, and power analysis were written in Intel Fortran 90/95. The interface and core modules for setting parameters, viewing results and drawing figures were written in JAVA. The software runs on Microsoft Windows XP/Vista/7/10/11. GAHP is an integrated and project-based software package. When the software is initiated, the first thing to do is to create a new project or open an existing project. The use of project will assure that all operations and running results are properly saved when the software is closed. When the project is open the next time by the software, previous operations and results can be recovered. Introduced below are the four functionalities implemented in the current version of GAHP.

### 2.4 The MHP functionality

Functionality MHP displays the completed linkage maps in a format (or style) which can be easily modified by users. Linkage maps should have been built by other software packages. Chromosome information and marker positions have to be provided. The input file for MHP consists of three parts: 1) general information on linkage maps, 2) marker number information, and 3) linkage map information. The example given in Supplementary Figure S1 represents a linkage map with seven chromosomes. Markers on their chromosomes were defined by marker interval, i.e. distance between adjacent markers in cM (Supplementary Figure S1A). Marker number on each chromosome and linkage map information are given in Supplementary Figures S1B and S1C, respectively.

**FIGURE 2:**
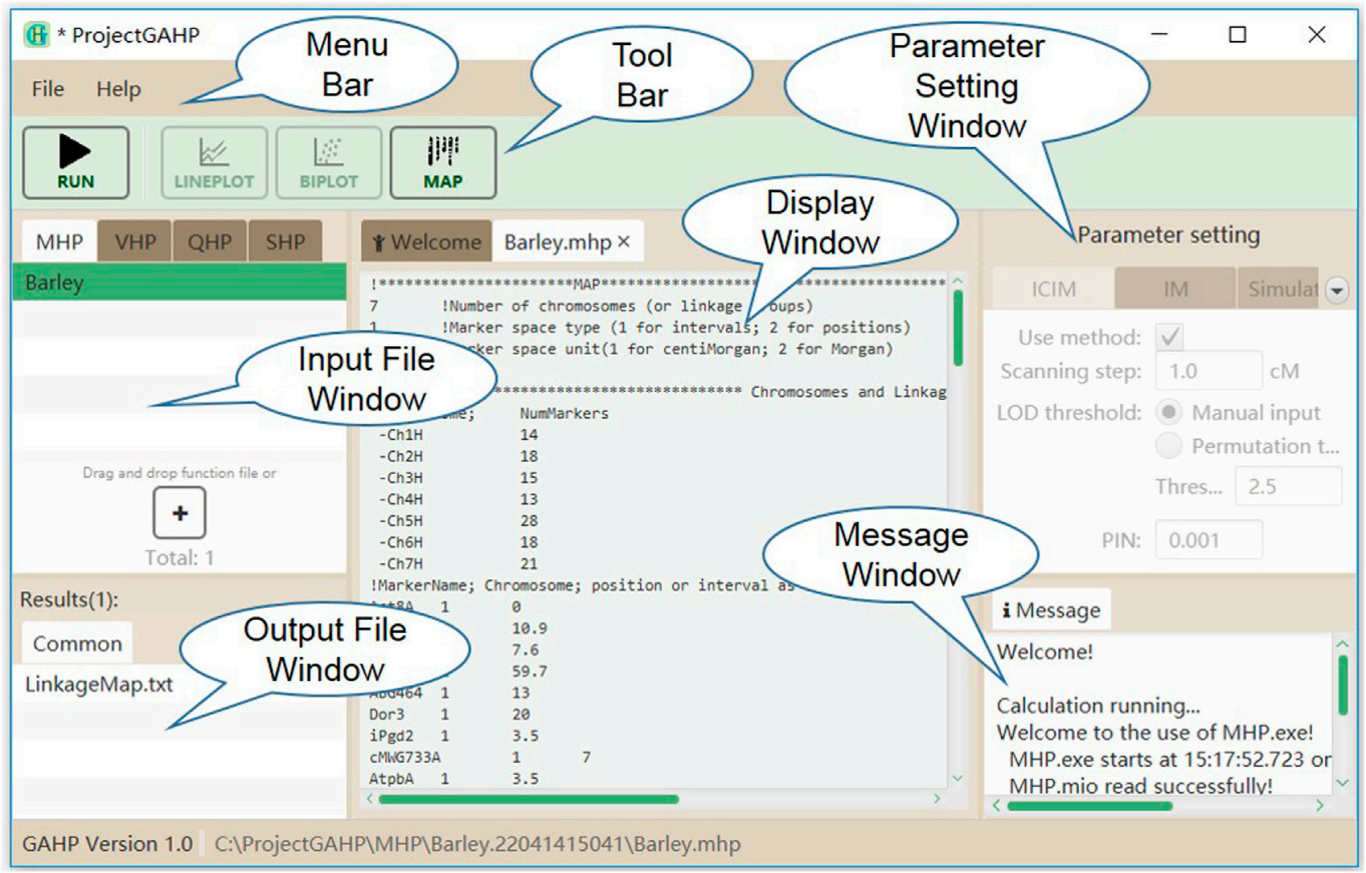
The interface of functionality MHP.

Figure 2 shows the interface of functionality MHP. The menu and tool bars are located on the top of the interface. The input and output file windows are located on the left side, showing names of the loaded input files and associated output files. In the input file window, files are grouped by functionalities, i.e. MHP, VHP, QHP, and SHP. In the output file window, files are grouped by population names, i.e. PIL, IB1, IB2 and IF2 *etc.* In the middle is the display window, which shows the detailed information of input or output files. At the right side are the parameter setting and running message windows. No parameter is needed to run functionality MHP. While the input file is properly loaded, the users may click “Run” on the tool bar to run the functionality.

### 2.5 The VHP functionality

Heritability may be the most important concept in quantitative genetics, which quantifies the proportion of genetic variation included in phenotypic values. Analysis of variance (ANOVA) can be used to estimate the variance components, based on which the broad-sense heritability can be estimated in genetic populations. Here the mapping populations can be some or all of the four populations as shown in Figure 1. Combined ANOVA will be applied if more than one population is included in the input file. The input file for VHP consists of five parts: 1) general information of the genetic populations, 2) phenotype of PIL, 3) phenotype of IB1, 4) phenotype of IB2, and 5) phenotype of IF2. If one population has no phenotypic data, the corresponding part in the input file is left to be empty. Supplementary Figure S2 represents an example of input file for VHP, where all the four populations have phenotypic values. Population sizes of PIL, IB1, IB2 and IF2 are equal to 200, 200, 200, and 300, respectively (Supplementary Figure S2A). Phenotypic values of the four populations were defined in Supplementary Figures S2B–S2E, respectively. It should be noted that populations IB1 and IB2 must have the same size as PIL, if included.

**FIGURE 3:**
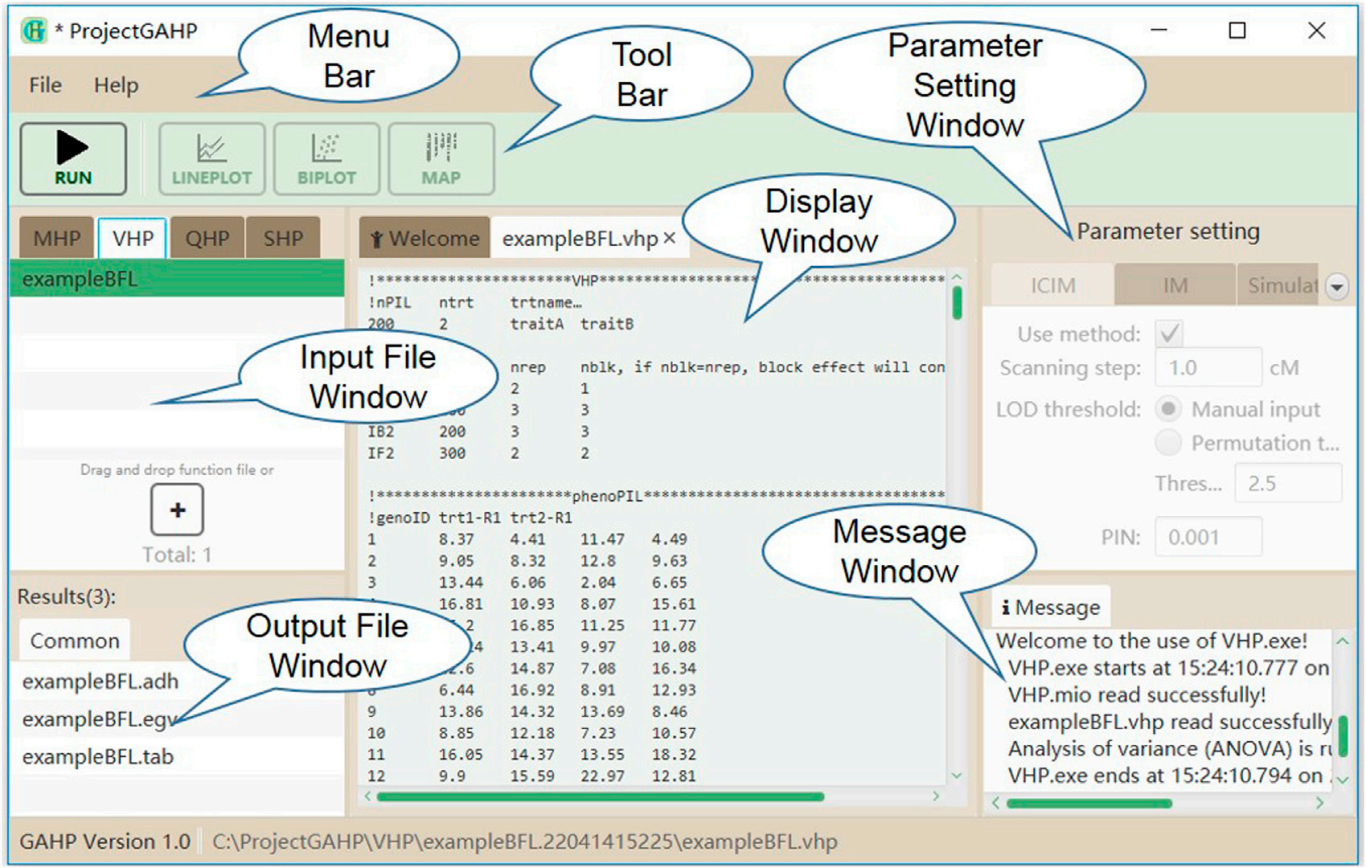
The interface of functionality VHP.

Figure 3 shows the interface of functionality VHP. Input files for this functionality are grouped on the VHP tab in the input file window. No parameter is needed to run this functionality. While the input file is properly loaded, the users may click “Run” on the tool bar to run the functionality.

### 2.6 The QHP functionality

As many as four populations, i.e. PIL, IB1, IB2, and IF2, can be used in QTL mapping either independently or together in functionality QHP, depending on the populations available. Firstly, the included populations are analyzed independently. Independent analysis is named by the respective population. Secondly, combined analysis is conducted using the included populations as many as possible. Names of the combined analysis are given in Table 1. Combined analysis using populations IB1 and IB2 is named by IBC; using populations IF2 and PIL is named by IFL; using populations IB1, IB2, and PIL is named by IBL; using populations IB1, IB2, and IF2 is named by IBF; and using populations IB1, IB2, IF2 and PIL is named by BFL (Table 1). The input file for QHP is composed of eight parts: 1) general information of mapping populations, 2) marker number information, 3) linkage map information, 4) marker types of PIL, 5) phenotype of PIL, 6) phenotype of IB1, 7) phenotype of IB2, and 8) phenotype of IF2. If one population has no phenotypic data, the corresponding part in the input file is left to be empty.

**TABLE 1:** Naming of the combined QTL mapping in functionalities QHP and SHP.

Combined analysis	Populations needed
IBC	IB1 and IB2
IFL	IF2 and PIL
IBL	IB1, IB2 and PIL
IBF	IB1, IB2 and IF2
BFL	IB1, IB2, IF2 and PIL

Supplementary Figure S3 represents an example of input file for QHP, where all the four populations have phenotypic values. Eleven parameters are included in general information (Supplementary Figure S3A): (1) type of pure lines in PIL, i.e. 1 for DHs, and 2 for RILs; (2) size of PIL in genotyping, i.e. number of genotyped pure lines in PIL (denoted as gPIL); (3) number of chromosomes or linkage groups; (4) mapping function, i.e. 1 for Kosambi’s function, 2 for Haldane’s function, and 3 for Morgan’s function; (5) marker space type, i.e. 1 for marker positions, and 2 for marker intervals; (6) marker space unit, i.e. 1 for centi-Morgan, and 2 for Morgan; (7) size of PIL in phenotyping; (8) size of IB1 in phenotyping; (9) size of IB2 in phenotyping; (10) size of IF2 in phenotyping; and (11) number of traits, followed by name of each trait. Population sizes of PIL, IB1, IB2 and IF2 in the example as given in Supplementary Figure S3A were equal to 200, 200, 200, and 300, respectively. Kosambi’s mapping function was used to convert recombination frequency to marker distance. Markers on the seven chromosomes were defined by positions. The unit of marker space was cM, and the number of phenotypic traits was equal to 1, named by simuTait. Marker number and linkage map information were given in Supplementary Figures S3B and S3C, respectively. Genotypic data at all polymorphic markers for all pure lines in PIL was given in Supplementary Figure S3D. Phenotypic values of the four populations were given in Supplementary Figures 3E–3H, respectively. As for functionality QHP, sizes of populations PIL, IB1, and IB2 have to be equal, if included.

**FIGURE 4:**
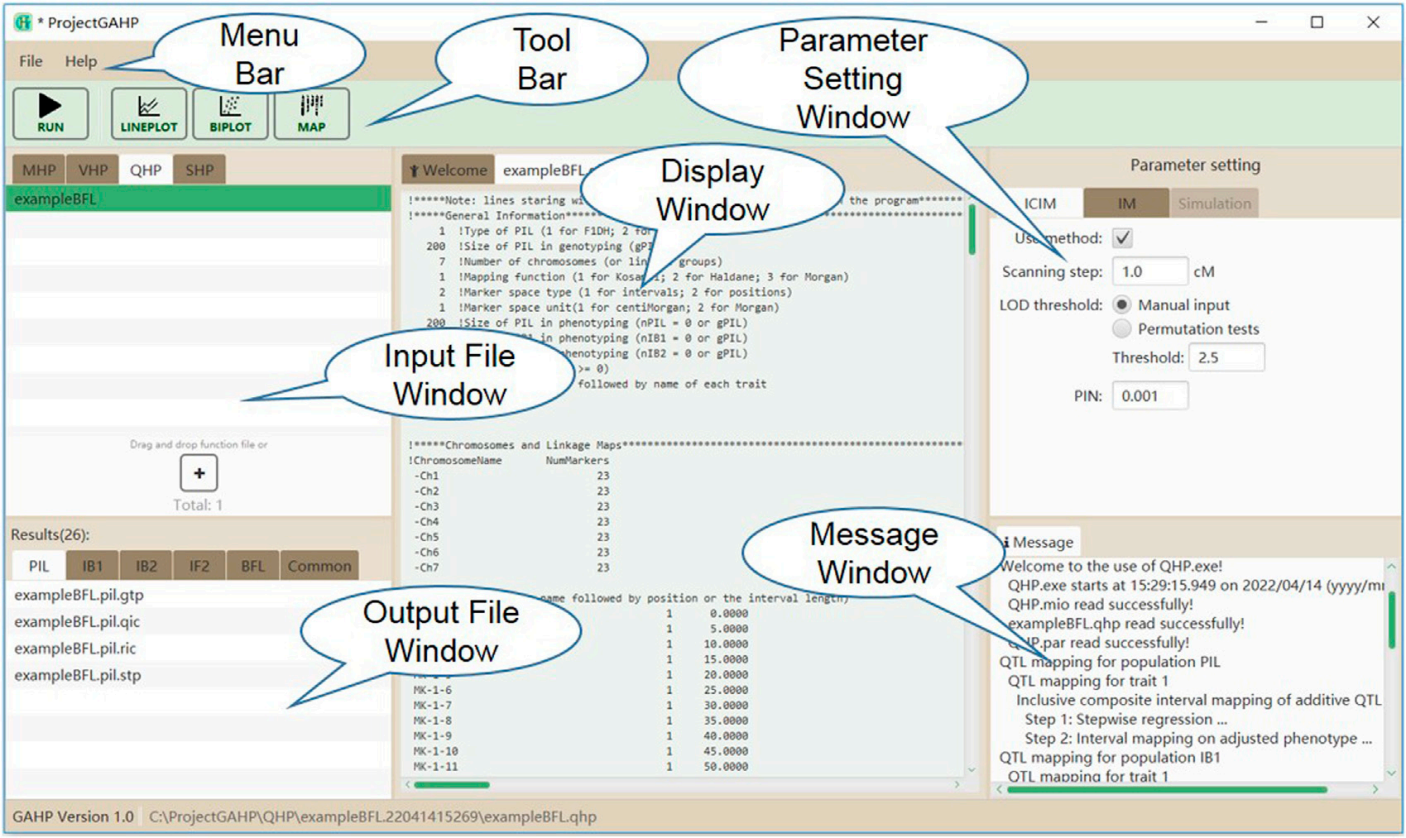
The interface of functionality QHP.

Figure 4 shows the interface of functionality QHP. Input files are grouped on the QHP tab in the input file window. Mapping parameters can be set in the parameter setting window, located at the right side of the interface. Two mapping methods are available in QHP, i.e., 1) IM: the conventional interval mapping for additive and dominant QTLs (Lander and Botstein, 1989); 2) ICIM: inclusive composite interval mapping for additive and dominant QTLs (Li et al., 2007; Zhang et al., 2008). After the mapping method selection and parameter setting, the users may click the “Run” button in the tool bar to run the functionality. Mapping results will be listed in the output file window, when the functionality is completed successfully.

### 2.7 The SHP functionality

In functionality SHP, populations PIL, IB1, IB2 and IF2 are generated for a set of predefined QTLs, and then power analysis is conducted on the simulated populations. Similar to functionality QHP, mapping methods IM and ICIM are provided in SHP. QTL mapping can be conducted in individual populations, as well as in multiple populations by combined analysis. Only one trait can be defined and simulated in one input file. The input file for SHP is composed of five parts: 1) general information of mapping populations, 2) marker number information, 3) linkage map information, 4) gene or QTL information, and 5) genotypic values of the predefined QTLs.

Supplementary Figure S4 represents an example input file to run functionality SHP, where all the four populations are simulated for power analysis. Thirteen parameters are included in general information of populations (Supplementary Figure S4A). The first ten parameters are same as those in functionality QHP. The other parameters are: (11) sampling PIL to generate IF2, i.e. 1 for random sampling, and 2 for sampling method that each line in PIL appears the same times in IF2; (12) indicator to define the content of the next parameter, i.e. 1 for heritability, and 2 for error variance; (13) heritability or error variance depending on the previous indicator, where F_2_ is used as the reference population to convert between heritability and error variance. Name of each chromosome and number of markers on the chromosome are specified first (Supplementary Figure S4B), followed by the definition of each chromosome (Supplementary Figure S4C). Each chromosome is defined by all markers located on, and the marker positions. The fourth part provides the number of QTLs or genes and their positions on each chromosome (Supplementary Figure S4D), and the fifth part provides the genotypic values of additive-dominant QTLs and epistatic networks (Supplementary Figure S4E).

**FIGURE 5:**
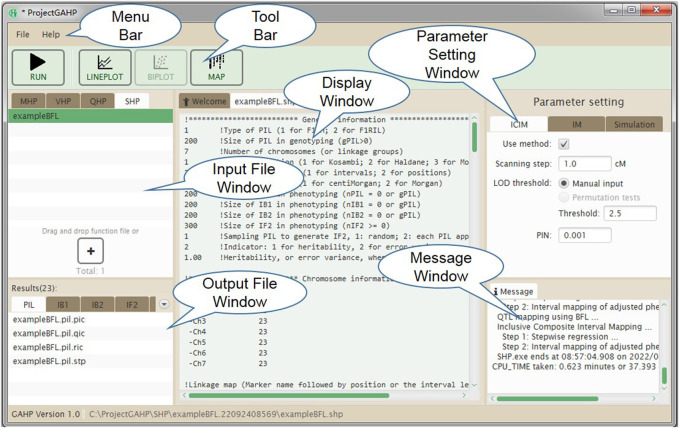
The interface of functionality SHP.

Figure 5 shows the interface of functionality SHP. Input files are grouped on the SHP tab in the input file window. In addition to the parameters for mapping methods (similar to functionality QHP), those for the simulation purpose also need to be specified in the parameter setting window, including random seed, number of runs, indicator whether or not to output the simulated populations, and support interval in cM for counting the true and false QTLs detected in simulated populations. After mapping method selection and parameter setting, the users may click the “Run” button in the tool bar to conduct the population simulation and QTL detection power analysis.

## 3 Results

### 3.1 Outputs of the MHP functionality

For the four functionalities implemented in the current version of GAHP, most output files have the same prefix name as the input file but with different extension names. Output file with extension name ‘*.txt’ is pure-text, providing the connection between interface and calculation kernel. There is only one output file after running MHP, named by ‘LinkageMap.txt’ (see the “common” tab in output file window in Figure 2), which contains the information of linkage maps given in the input file. GAHP provides the user-friendly interface to draw the linkage maps for individual chromosomes (Supplementary Figure S5A), or all chromosomes simultaneously (Supplementary Figure S5B). Options are provided for users to change the style of map drawing, including the position label, marker name, separator line, chromosome height, number of chromosomes per row, and gradient color.

### 3.2 Outputs of the VHP functionality

Three output files are generated after running the VHP functionality (see the “common” tab in output file window in Figure 3). Output with extension name ‘*.adh’ contains the estimates of variance components and heritability (Supplementary Figure S6). The first part provides the estimates of genotypic variance (Vgeno), error variance (Verror), phenotypic variance (Vpheno), heritability in the broad sense (Hbroad), and degree of freedom of random error (DFerror) for each trait in each population. The second part provides the estimates of additive variance (Vadd_F2), dominant variance (Vdom_F2), error variance (Verror_F2), heritability in the narrow sense (Hnarrow_F2), and degree of freedom of random error (DFerror) from the combined ANOVA using all populations, where F_2_ is assumed to be the reference population. Output with extension name ‘*.egv’ contains the estimated genotypic value of each line in population PIL or each hybrid in populations IB1, IB2 and IF2 for each trait (Supplementary Figure S7). Output with extension name ‘*.tab’ contains the conventional ANOVA table for each trait. As an example, Supplementary Figure S8 shows ANOVA tables of two traits in population PIL. All populations included in input files have their corresponding ANOVA tables in this output file.

### 3.3 Outputs of the QHP functionality

QHP is the key functionality in GAHP. Outputting results are grouped by names of individual population (i.e. PIL, IB1, IB2, or IF2) and combined QTL mapping (i.e. IBC, IFL, IBL, IBF, or BFL; see the lower left window in Figure 4). For output files arranged in each group, three lower case characters after the prefix indicate the group name, i.e. ‘*.pil’, ‘*.ib1’, ‘*.ib2’, ‘*.if2’, ‘*.ibc’, ‘*.ifl’, ‘*.ibl’, ‘*.ibf’, or ‘*.bfl’. The last three lower case characters are the extension name, indicating contents in each output. Each mapping method (i.e. IM, and ICIM) has three kinds of outputting information, which are labeled by Q for detected QTLs, R for results at every scanning position, and T for permutation tests (Table 2). For ICIM, two additional output files with extension names ‘*.stp’ and ‘*.gtp’ are provided, containing the results from stepwise regression, and the predicted genotypes at each detected QTL and genotypic values, respectively. As many as four mapping populations can be included, and thus there may be at most five groups of ‘*.stp’, ‘*.gtp’, Q, R and T output files, four for independent population mapping, and one for combined QTL mapping. As an example, Supplementary Figure S9 gives part of the content in output ‘*.bfl.ric’ from simulated populations, i.e., mapping results from ICIM in combined mapping BFL (denoted as BFL-ICIM) at each scanning position; Supplementary Figure S10 gives the content in output ‘*.bfl.qic’ from ICIM, i.e., information of the detected QTLs. For each QTL, the chromosomal position, nearest left marker, nearest right marker, total LOD score, LOD score for additive effect, LOD score for dominant effect, total phenotypic variance explained (PVE), additive PVE, dominant PVE, additive effect, dominant effect, and one-LOD confidence interval are reported.

**TABLE 2:** Description of output files from the QHP functionality.

Group	Extension name	Description of contents
Results related to individual population or combined QTL mapping	STP	Selected marker variables and their effects from the first step of stepwise regression in inclusive composite interval mapping (ICIM)
	QIM, QIC	QTL identified from interval mapping (IM), and ICIM
	RIM, RIC	Results at every one-dimensional scanning position from IM and ICIM
	TIM, TIC	LOD score from permutation tests for IM and ICIM
	GTP	Bayesian classification of genotypes at QTLs identified from ICIM
Common, i.e. results not related to QTL mapping	COE	Lower triangular matrix of pairwise correlation coefficient between markers in population PIL
	MTP	Frequency of marker types, Chi-square test for segregation distortion, and missing-imputed marker types
	STA	Descriptive statistics of phenotypes
	TXT	Three text files, i.e. ‘LinkageMap.txt’, ‘Phenotype.txt’ and ‘Threshold.txt’, are used for the connection between interface and QTL mapping kernel

Outputs not related to QTL mapping are listed under the ‘Common’ group (see the lower left window in Figure 4). There are six such output files recording the relevant information in mapping populations (Table 2). Output with extension name ‘*.coe’ contains the pair-wise correlation coefficients between markers in population PIL, which may be used to check the quality of linkage maps. Output with extension name ‘*.mtp’ contains marker summary, and marker types after the imputation of missing values. Output with extension name ‘*.sta’ contains the descriptive statistics of phenotypic values in each population. Three text files, i.e. ‘LinkageMap.txt’, ‘Phenotype.txt’ and ‘Threshold.txt’ contain information of the linkage map, phenotypic values, and threshold LOD score, respectively, which are used for the connection between interface and QTL mapping kernel.

**FIGURE 6:**
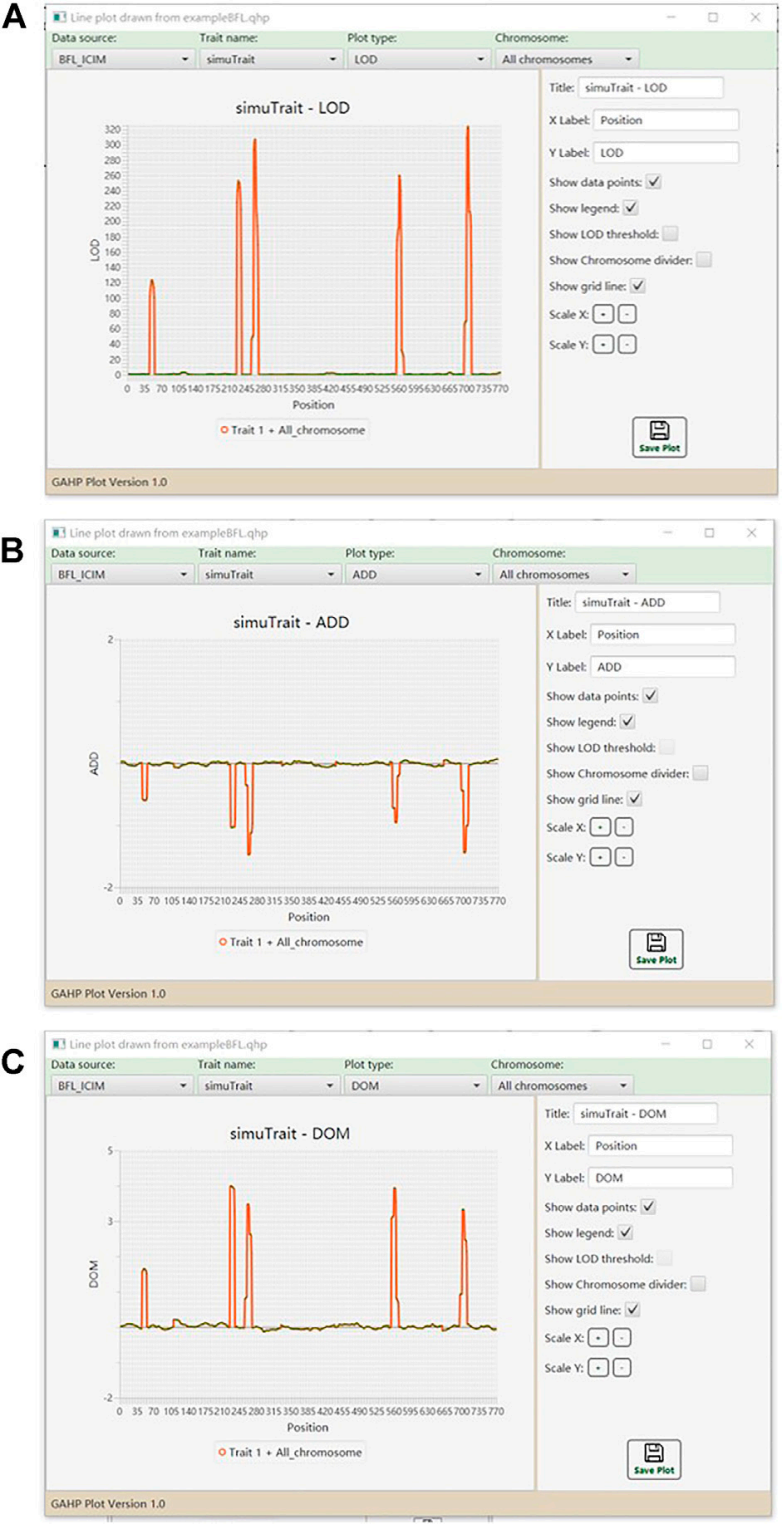
Line plots for QTL mapping results. **(A)** LOD score. **(B)** Additive effect. **(C)** Dominant effect.

Graphs of LOD score and genetic effects on each chromosome or on all chromosomes are available in the QHP functionality. Figure 6 shows the one-dimensional profile of LOD score, additive and dominant effects on one trait in simulated populations from BFL-ICIM. Tool bars are provided for the users to select the source of data, and modify the parameters so as to change the style of graphs. Bi-plot graphs for phenotypic data are also available. For example, Supplementary Figure S11 shows the bi-plot for phenotypic data of individuals in population IF2 together with their mid-parental values.

### 3.4 Outputs of the SHP functionality

Similar to QHP, outputting results from functionality SHP are also grouped by names of individual population and combined QTL mapping (see the lower left window in Figure 5). For output files arranged in each group, three lower case characters after the prefix indicate the group name. The last three lower case characters are the extension name, indicating contents in each output. Each mapping method (i.e. IM, and ICIM) generates three kinds of output files, labeled by Q for detected QTLs, R for results at all scanning positions, and P for power analysis (Table 3). Output file ‘*.stp’ is generated only for ICIM. There may be at most five groups of ‘*.stp’, Q, R and P files, four for individual population mapping, and one for combined QTL mapping. By looking into the P output files, the users can compare the QTL detection power from different mapping methods. Formats of the Q and R outputs are similar to those from the QHP functionality, but the Q output files in SHP contain the detected QTLs from each simulation run, and the R output files in SHP contain the average LOD score and effects across all simulation runs. Supplementary Figure S12 gives part of the content in output file ‘*.bfl.pic’ from an example input file. The first part contains the detection power, LOD score and estimated effects from ICIM for each QTL in simulation, and the second part contains the corresponding information for each marker interval.

**TABLE 3:** Description of output files from the SHP functionality.

Group	Extension name	Description of contents
Results related to individual population or combined QTL mapping	STP	Selected marker variables and their effects from the first step of stepwise regression in inclusive composite interval mapping (ICIM) for each simulation run
	QIM, QIC	QTL identified from interval mapping (IM), and ICIM
	RIM, RIC	Results at all one-dimensional scanning positions from IM and ICIM
	PIM, PIC	Power of predefined QTLs together with false positives from IM and ICIM
Common, i.e. results not related to QTL mapping	TXT	Two text files, i.e. ‘LinkageMap.txt’, and ‘Threshold.txt’, are used for the connection between interface and QTL mapping kernels
	GMD	Input file for the Blib simulation platform, which defines the genetic model on the simulated trait
	QHP (optional)	Simulated populations in the format that can be directly loaded to functionality QHP

Outputs not related to QTL mapping are listed under the ‘Common’ group (see the lower left window in Figure 5). One output has the name ‘SHP.gmd’, which is arranged in a format that can be directly used as the input of the Blib platform of genetics and breeding simulation, i.e., genetic model of the simulated trait (Table 3). Two text files, i.e. ‘LinkageMap.txt’ and ‘Threshold.txt’ contain information of the linkage map and threshold LOD score. If the check box “Outputting population” in the parameter setting window is clicked, the simulated populations are arranged in the format that can be directly used as input files for the QHP functionality.

SHP also provides the graphic option of LOD scores and genetic effects on one chromosome or on all chromosomes, averaged from all simulation runs, which are similar to functionality QHP.

## 4 Discussion

### 4.1 Applications of the GAHP software package in genetic studies

Heterozygous populations are needed in order to investigate the dominance-related genetic effects, which are critical to understanding the genetic mechanism of heterosis in plants. Conventional bi-parental F_2_ are such populations, but have the disadvantage in conducting the multi-environmental and replicated phenotyping trials. As one replacement, immortalized F_2_ populations can overcome the disadvantage and provide the estimates of additive, dominant and epistatic effects. In addition, genotyping is only needed on pure lines in population PIL, which are the direct parents of F_1_ hybrids consisting of the immortalized population (Hua et al., 2003; Liu et al., 2020). Immortalized BC population with one parental line has only two genotypes at each locus, and therefore cannot provide the full information to estimate the dominant effect. However, when used together, immortalized BC populations at both directions to the original two parental lines can also be used in investigating the genetic basis of heterosis (Li et al., 2008b; Aakanksha et al., 2021).

GAHP is freely available from https://isbreeding.caas.cn. Users’ manual and sample datasets are automatically included when the package is properly installed in local personal computers. A video tutorial is provided on the software webpage. GAHP can conduct the phenotypic data analysis, and QTL mapping on pure-line populations and their derived immortalized BC and F_2_ populations, either separately or in combination. Both additive and dominant variances can be estimated by the combined ANOVA in the SHP functionality, by which the broad-sense and narrow-sense heritabilities can be calculated. Both additive and dominant effects of QTLs can be estimated by the combined QTL mapping on immortalized BC and F_2_ populations in the QHP functionality. Combined mapping utilizes more populations, and improves the estimation accuracy of genetic variances, heritabilities, and positions and effects of QTLs. In addition, GAHP can simulate the four kinds of mapping populations (Figure 1), based on the user-defined information on linkage map, QTL locations and effects, and error variance (or heritability). Mapping results from the simulated populations allow the users to investigate of efficiency of genetic studies on immortalized populations. Furthermore, the SHP functionality in GAHP allows a perspective comparison of mapping methods through power analysis. QTL detection power is affected by many factors, such as population size, heritability of phenotypic trait, QTL locations and effects, marker density, and the linkage relationship between QTLs (Li et al., 2010). Evaluation of mapping methods can be based on QTL detection power and false discovery rate (FDR). A better mapping method in the sense of statistics should have higher detection power and lower FDR (Li et al., 2010). The SHP functionality provides an approach to comparing the mapping methods in immortalized populations by considering the factors affecting mapping efficiency. SHP can also be used to investigate the efficiency of combined analysis using different populations, effect of population size on QTL detection, and various crossing schemes in PIL to generate the IF2 population *etc.* When new mapping methods are developed, the simulated populations generated by SHP can be used to evaluate their efficiency.

### 4.2 Features of the GAHP integrated package

In most QTL mapping packages, only the independent population analysis is provided, such as QTL IciMapping (Meng et al., 2015), GACD (Zhang et al., 2015b) and GAPL (Zhang et al., 2019). The four kinds of populations that can be handled in GAHP are highly related (Figure 1), which provides the opportunity for combined analysis. Mapping accuracy of independent population in the QHP functionality is actually the same as the BIP functionality in QTL IciMapping (Li et al., 2007; Zhang et al., 2008; Meng et al., 2015). It is expected that the combined QTL mapping in QHP on multiple populations should provide more accurate estimation on QTL positions and effects. Functionality AOV in QTL IciMapping (Meng et al., 2015) and VHP in GAHP are both developed for phenotypic ANOVA and heritability estimation. AOV in QTL IciMapping is suitable for individual populations phenotyped in single-environmental or multi-environmental trials, by which only the broad-sense heritability can be estimated. VHP in GAHP is specifically designed for the four related populations as shown in Figure 1, by which both broad-sense and narrow-sense heritabilities can be estimated, since the additive and dominant variances can be separated by the combined ANOVA across populations. It should be noted that only the phenotypic values from single-environmental trials are acceptable in the current version of GAHP.

Linkage map used in functionality QHP is based on genotypes of pure lines in population PIL, which should be constructed by other software packages, such as QTL IciMapping (Meng et al., 2015; Zhang et al., 2020). There is no need to rebuild the linkage maps in immortalized BC or F_2_ populations. Therefore, map construction is not considered in GAHP. Instead, functionality MHP is developed in GAHP to display the completed linkage maps. MHP can handle larger number of markers and make higher quality of linkage maps, in comparison with QTL IciMapping. In input files of functionality QHP, genotypes are only needed for population PIL; genotypes of hybrids in immortalized BC and F_2_ populations can be derived from pure lines and two original inbred parents. When using functionalities VHP and QHP, it is expected that the phenotypic values of different populations are collected in the same environment so as to avoid the effect of environments and genotype by environment interactions.

Time spent in QTL mapping should be taken into consideration when a large number of markers are included. When populations PIL, IB1, IB2 and IF2 are fixed at a size of 1000, the time spent for SHP to complete one simulation run was around 1, 12 and 55 min for marker numbers 200, 2000 and 20000, respectively. The time spent in one run was to complete four independent population analysis, and one combined analysis. The time spent for independent population analysis was close to that in QTL IciMapping for the same dataset. The time spent for combined analysis is slightly longer than that for independent population. The current version of GAHP can handle a number of markers as much as 50000. In most bi-parental populations, number of polymorphic markers may be much smaller than 50000. When more markers are included, binning analysis can be conducted to reduce the marker number and running time.

### 4.3 Further refinement of the GAHP package

At present, only one-dimensional QTL mapping is available in GAHP. In addition to additive and dominant effects, epistasis is also an important source of variation of complex traits, which maintains the additive variance and assures the long-term genetic gain in breeding (Zhang et al., 2012). Epistasis plays an important role in genetic basis of heterosis as well (Hua et al., 2003). QTL by environment interaction (QEI) widely exists in plants. Studies on epistasis and QEI contribute to the better understanding of genetic architecture of quantitative traits and heterosis (Li et al., 2015; Liu et al., 2020). It can be imagined that the algorithms of epistatic and QEI mapping would be more complicated than that of additive and dominant mapping in one environment. Nevertheless, ICIM has been extended to epistatic and QEI mapping in bi-parental populations (Zhang et al., 2012; Li et al., 2015). In the future, we may consider the extension of ICIM to epistatic and QEI mapping using multiple immortalized populations, and implement the mapping algorithms in GAHP. In addition, heterosis can also be studied by diversity inbred lines and their F_1_ hybrids obtained by suitable crossing designs. The hybrid population derived from a diversity of inbred lines has different structure from population IF2 as discussed in this study, which may require further studies on genetic analysis method. Once developed and validated, the analysis method can be added as a separate functionality to extend the applications of GAHP in genetic studies.

## Data Availability

The original contributions presented in the study are included in the article/Supplementary Material, further inquiries can be directed to the corresponding authors.
