# Supplementary material for: GAHP: An integrated software package on genetic analysis with bi-parental immortalized heterozygous populations
Source: Front Genet. 2022 Oct 5;13:1021178. doi: 10.3389/fgene.2022.1021178 (PMC9579317; doi:10.3389/fgene.2022.1021178)
Supplement: Supplementary file 1 [file Table1.docx]

Supplementary Material

# Supplementary Figures

**Supplementary Figure 1.** The MHP input file in the EXCEL with four sheets

**Supplementary Figure 2.** The VHP input file in the EXCEL with five sheets

**Supplementary Figure 3.** The QHP input file in the EXCEL with eight sheets

**Supplementary Figure 4.** The SHP input file in the EXCEL with five sheets

**Supplementary Figure 5.** Linkage map for one chromosome or all chromosomes

**Supplementary Figure 6.** Information of the ADH file from the VHP functionality

**Supplementary Figure 7.** Information of the EGV file from the VHP functionality

**Supplementary Figure 8.** Information of the TAB file from the VHP functionality

**Supplementary Figure 9.** Information of the BFL.RIC file from the QHP functionality

**Supplementary Figure 10.** Information of the BFL.QIC file from the QHP functionality

**Supplementary Figure 11.** Bi-plots for phenotypic data

Figure S12

**Supplementary Figure 12.** Information of the BFL.PIC file from the SHP functionality
